# Genome profiling of *ERBB2*-amplified breast cancers

**DOI:** 10.1186/1471-2407-10-539

**Published:** 2010-10-08

**Authors:** Fabrice Sircoulomb, Ismahane Bekhouche, Pascal Finetti, José Adélaïde, Azza Ben Hamida, Julien Bonansea, Stéphane Raynaud, Charlène Innocenti, Emmanuelle Charafe-Jauffret, Carole Tarpin, Farhat Ben Ayed, Patrice Viens, Jocelyne Jacquemier, François Bertucci, Daniel Birnbaum, Max Chaffanet

**Affiliations:** 1 Marseille Cancer Research Center, UMR891 Inserm, Institut Paoli-Calmettes, Department of Molecular Oncology, Marseille, France; 2 Department of BioPathology, Institut Paoli-Calmettes, Marseille, France; 3 Department of Medical Oncology, Institut Paoli-Calmettes, Marseille, France; 4 Université de la Méditerranée, Marseille, France; 5 Department of Medical Oncology, Salah Azaiz Institute, Tunis, Tunisia

## Abstract

**Background:**

Around 20% of breast cancers (BC) show *ERBB2 *gene amplification and overexpression of the ERBB2 tyrosine kinase receptor. They are associated with a poor prognosis but can benefit from targeted therapy. A better knowledge of these BCs, genomically and biologically heterogeneous, may help understand their behavior and design new therapeutic strategies.

**Methods:**

We defined the high resolution genome and gene expression profiles of 54 *ERBB2*-amplified BCs using 244K oligonucleotide array-comparative genomic hybridization and whole-genome DNA microarrays. Expression of ERBB2, phosphorylated ERBB2, EGFR, IGF1R and FOXA1 proteins was assessed by immunohistochemistry to evaluate the functional ERBB2 status and identify co-expressions.

**Results:**

First, we identified the *ERBB2*-*C17orf37*-*GRB7 *genomic segment as the minimal common 17q12-q21 amplicon, and *CRKRS *and *IKZF3 *as the most frequent centromeric and telomeric amplicon borders, respectively. Second, GISTIC analysis identified 17 other genome regions affected by copy number aberration (CNA) (amplifications, gains, losses). The expression of 37 genes of these regions was deregulated. Third, two types of heterogeneity were observed in *ERBB2*-amplified BCs. The genomic profiles of estrogen receptor-postive (ER+) and negative (ER-) *ERBB2*-amplified BCs were different. The WNT/β-catenin signaling pathway was involved in ER- *ERBB2*-amplified BCs, and *PVT1 *and *TRPS1 *were candidate oncogenes associated with ER+ *ERBB2*-amplified BCs. The size of the *ERBB2 *amplicon was different in inflammatory (IBC) and non-inflammatory BCs. *ERBB2*-amplified IBCs were characterized by the downregulated and upregulated mRNA expression of ten and two genes in proportion to CNA, respectively. IHC results showed (i) a linear relationship between *ERBB2 *gene amplification and its gene and protein expressions with a good correlation between ERBB2 expression and phosphorylation status; (ii) a potential signaling cross-talk between EGFR or IGF1R and ERBB2, which could influence response of *ERBB2*-positive BCs to inhibitors. FOXA1 was frequently coexpressed with ERBB2 but its expression did not impact on the outcome of patients with *ERBB2*-amplified tumors.

**Conclusion:**

We have shown that ER+ and ER- *ERBB2*-amplified BCs are different, distinguished *ERBB2 *amplicons in IBC and non-IBC, and identified genomic features that may be useful in the design of alternative therapeutical strategies.

## Background

Gene amplification is a frequent alteration in breast cancer (BC) that affects multiple genomic regions [1-3]. One of the most studied amplifications is located in chromosomal region 17q12 and involves the *ERBB2 *gene. *ERBB2 *encodes a transmembrane tyrosine kinase receptor of the ERBB/EGFR family. *ERBB2 *is amplified in around 20% of BCs. The receptor is overexpressed in most amplified cases and in some non-amplified cases as well. This alteration is associated with a poor clinical outcome. BCs with ERBB2 overexpression can benefit from a targeted therapy that uses the humanized monoclonal antibody trastuzumab or the ERBB kinase inhibitor lapatinib [4,5]. However, resistance to trastuzumab is frequent [6] and its mechanisms are poorly understood [7], although ERBB2 phosphorylation [8], PTEN and PIK3CA status [9] seem important factors.

*ERBB2 *gene amplification can be detected by various methods including fluorescence *in situ *hybridization (FISH) or quantitative PCR [10]. Overexpression of the receptor is detected by immunohistochemistry (IHC) by using the standardized Dako Herceptest. Gene expression studies have shown that BCs with a specific gene expression signature including *ERBB2*-overexpression constitute a separate molecular subtype [11-13]. However, a substantial number of breast tumors assigned to the ERBB2 subtype lacks ERBB2 protein expression and/or *ERBB2 *gene amplification [14,15] and ERBB2-positive cancers that express estrogen receptor (ER) fall into the luminal subtypes [11,13,16].

Several 17q12-q21 genes are variably coamplified and coexpressed with *ERBB2 *[17]. This could influence the response to trastuzumab and/or constitute accessory targets for synergistic treatment [18-21]. A better knowledge of *ERBB2-*amplified BCs may thus help design new therapeutic strategies. To better characterize this particular group of BCs, we used high-resolution array-comparative genomic hybridization (aCGH) and whole-genome DNA microarrays to define the genome and gene expression profiles of 54 BCs with *ERBB2 *amplification.

## Methods

### Breast cancer samples

Tumor tissues were collected from 340 patients with invasive adenocarcinoma who underwent initial surgery at the Institut Paoli-Calmettes (Marseille, France) between 1987 and 2007 (from a cohort of 2,175 patients with frozen tumor sample) and from a series of 91 Tunisian T4d tumors (TNM, UICC) treated between 1994 and 1998 at the Salah Azaiz Institute (Tunis, Tunisia). Each patient gave informed consent and the study was approved by our institutional review board (also called "COS"). Samples were macrodissected and frozen in liquid nitrogen within 30 minutes of removal. The panel was not made of consecutive tumors but enriched in various forms of BCs. These include inflammatory BCs (IBCs), non-inflammatory BCs (NIBC), very young women BCs (YWBCs, ≤ 35 years) and older women BCs (OWBCs, ≥ 45 years).

The 340 tumors were analyzed by high resolution aCGH 244K in our previous studies [[3,22], unpublished data]. In all these cases, profiled samples were always collected before any systemic therapy (chemotherapy, hormone or trastuzumab therapy). They corresponded to a tumor surgically removed for NIBCs, and to the diagnostic surgical biopsy for IBCs. A total of 54 (16%) cases presented amplification of the *ERBB2 *locus. Features of these *ERBB2*-amplified tumors are reported in Additionnal file 1-Table S1. All specimens contained >60% of tumor cells (as assessed before RNA extraction using frozen sections adjacent to the profiled samples). IHC data included status for estrogen and progesterone receptors (ER and PR), P53 (positivity cut-off value of 1%), ERBB2 (0-3+ score, DAKO HercepTest kit scoring guidelines, defined as positive with 3+ and 2+ controled by FISH according to ASCO guidelines), and Ki67 (positivity cut-off value of 20%).

### Cell lines

Fourteen BC cell lines with *ERBB2 *amplification (BT-474, HCC202, HCC1569, HCC1954, HCC2218, JIMT-1, MDA-MB-361, MDA-MB-453, SK-BR-3, UACC-812, UACC-893, ZR-75-30 [23], SUM-190 and SUM-225 [24]) were grown as recommended by their supplier. These cell lines have been studied by FISH and QPCR and tested for trastuzumab response [8]. Most have also been previously studied by aCGH [25].

### DNA and RNA extraction

DNA and RNA were extracted from frozen samples by using guanidium isothiocynanate and CsCl gradient, as previously described [26]. RNA integrity was controled on Bioanalyzer (Agilent Technologies).

### Array-comparative genomic hybridization (aCGH) and expression data analyses

Genomic imbalances were determined by aCGH using 244K CGH oligonucleotide microarrays (Hu-244A, Agilent Technologies). Comparison of the results obtained with FISH and aCGH for several gene amplicons demonstrated a perfect agreement between the two techniques [3]. Gene expression data of 51 of the 54 BCs and 4 normal breast (NB) samples (NB1, NB2, NB3 and NB4, representing samples from 4 women and 3 commercial pools of respectively 1, 2 and 4 normal breast RNA, Clontech, Palo Alto, CA) were quantified by using whole-genome DNA microarrays (HG-U133 plus 2.0, Affymetrix). Experiments and data analyses were done as described [3]. Microarray data were deposited in Gene Expression Omnibus (GEO) under the following name: GSE 17907. To identify significant altered regions through the whole genome, we used the GISTIC algorithm to score the Copy-Number Genome across the 54 BCs [27] with a bootstrap procedure to calculate the significance level. Established for each genomic segment, this score takes into account the frequency of gene alterations (amplifications, gains, losses) and their gene copy number level. Expression data, up- and down- regulation were defined using normal breast expression comparison, with a |2-fold| threshold [3].

The gene expression signature (GES) associated with ER+ and ER- *ERBB2*-amplified BCs was obtained using Signal-to-Noise Ratio (SNR) and its significance was assessed using resampling with 100 permutations as described [28]. Unsupervised analysis was done with the Cluster program using log_2_-transformed data [29]. Expression data were median-centred on genes. For aCGH analysis, we evaluated robustness of clusters using multiscale bootstrap resampling; the robustness of each cluster was estimated with the R-package pvclust [30] using Ward's agglomerative method, Euclidean distance and 1000 bootstrap replications as parameters. Pvclust provides AU (approximately unbiased) p-values computed by multiscale bootstrap resampling. Cluster agglomerative parameters were centroid-linkage for expression [13]. Pearson correlation was used as similarity metrics for both of them.

Details concerning statistical methods are given in Additionnal file 2.

### Pathway/network analysis

The biologic relevance of the data was estimated using the Ingenuity Pathway Analysis (IPA) software (Ingenuity Systems) [31]. This functional annotation and network-mapping tool enables the discovery, visualization, and exploration of biologically and therapeutically relevant networks of gene interactions from the experimental data. Focus genes were imported using their Entrez Gene ID to be mapped to the Ingenuity database. The identified genes were mapped to genetic networks available in the Ingenuity database and were then ranked by the probability that a collection of genes equal to or greater than the number in a network could be achieved by chance alone. Confidence level of 99.9% was chosen as cut-off. Ingenuity^® ^software [31] was then used to identify canonical pathways and networks.

### Immunohistochemistry (IHC)

IHC was done using commercially available mouse monoclonal antibodies directed against human ERBB2, FOXA1, phosphorylated ERBB2 (pERBB2), IGF1R, and EGFR (Additionnal file 1-Table S2). The HercepTest (DAKO) is directed against the intracellular region of ERBB2 with high sensitivity; TAB250 anti-ERBB2 recognizes with high specificity but low sensitivity the extracellular region of the receptor [10]. Phosphorylated ERBB2 status was assessed using monoclonal mouse anti-human HER2-pY-1248 with CSA II biotin-free tyramide signal amplification system (DakoCytomation) as described [8].

IHC was done on 5-μm sections of tissue fixed in alcohol formalin for 24 hours and embedded in paraffin as described [32], using LSAB^R^2 kit in the autoimmunostainer (Dako Autostainer). According to a previous study [33], effects of fixation in this condition time would not affect the immunohistochemical detection of hormone receptors in breast cancer. The optimal titer for each antibody (except for HercepTest and G11 directed against ERBB2 and IGF1R, Additionnal file 1-Table S2) was established based on negative and positive controls. In addition, the dilution took into account the expected topography of the immunostaining (nucleus, cell membrane, and cytoplasm). After staining, slides were evaluated by two pathologists (J.J. and E.C.J.). Results for EGFR, pERBB2, FOXA1 and IGF1R status were scored by the quick score (QS) [32], taking into account the percentage (P) and intensity (I) of immunostaining (QS = P × I varies from 0 to 300) except for ERBB2 status (defined with ERBB2 TAB250 or HercepTest kit), which was evaluated with the Dako scale (0-3+, HercepTest kit scoring guidelines). Discrepancies were resolved under multiheaded microscope. We chose a uniform and clear cutoff value of QS > 0 for all antibodies, except for ERBB2 where the HercepTest scale was used: negative 0-1+ and positive 2+ and 3+.

### Statistical analyses

Associations of EGFR, ERBB2, pERBB2, FOXA1 and IGF1R expression with histoclinical features were assessed with Spearman's rank-correlation test or Mann-Whitney U test, as appropriate. Overall survival (OS) time was defined as the period from the date of starting first-line chemotherapy until the date of death from any cause or until the date of the last follow-up, at which point data were censored. Metastasis-free survival (MFS) was defined by the time interval between the diagnosis of BC and a distant metastasis. Metastasis-free patients were right censored at the date of the last follow-up, death, recurrence of local or regional disease, or development of a second primary cancer. Kaplan-Meier method was used to plot OS or MFS curves according to IHC results, and the significance of differences was assessed with the log-rank test. Univariate and multivariate Cox proportional hazard models were used to estimate the relations of protein expressions and clinical characteristics to OS. All reported p-values were two-sided, and the level of significance was set at p < 0.05. Variables for multivariate analysis were selected by means of a forward stepwise approach, using a significance level of p < 0.10 for entering into or remaining in the model. All analyses were done with the software package StatView, version 5.0 (SAS Institute, Inc.).

## Results

### Characterization of *ERBB2*-amplified BCs and cell lines

From our previous aCGH studies [[3,8,22], unpublished data], we identified 54 tumors and 14 cell lines exhibiting regional 17q12-q21-amplification (with a threshold log_2 _ratio >1) centered on *ERBB2 *(Additionnal file 3-Figure S1A-C and S1D, respectively). The *ERBB2 *frequency of amplification (16%, 54/340) observed in our panel was close to the frequency values obtained in previous studies [34-37].

**Figure 1:**
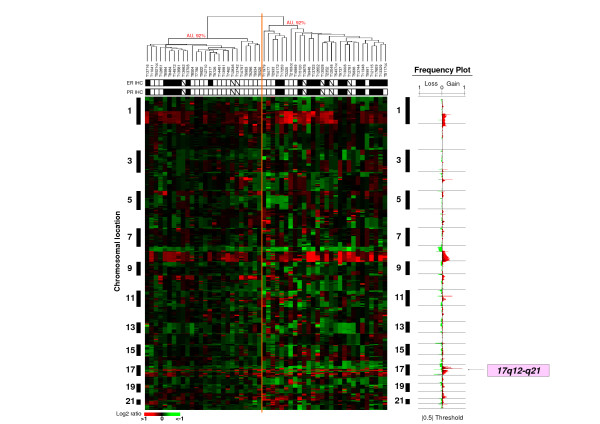
**aCGH profiling of *ERBB2*-amplifed BCs**. Unsupervised hierarchical clustering of genome copy number profiles measured for 54 *ERBB2*-amplified primary breast tumors by aCGH on 225,388 probes (without X and Y). Red indicates increased genome copy number and green indicates decreased genome copy number. The bars to the left indicate chromosome locations with chromosome 1pter to the top and 22qter to the bottom. The location of the odd-numbered chromosomes is indicated at left. The vertical orange dotted line defines the limits of the two major sample clusters defined by their respective AU (approximately unbiased) p-values Below the dendrogram, name of tumors are given and the rows indicate their ER and PR IHC status (black square, ER+ or PR+; white square, ER- or PR-). CNA frequencies discriminate ER+ and ER- *ERBB2-*amplified breast tumors (see also Additionnal file 1-Table S3). On the right, frequencies of CNA (gains and losses) are plotted as a function of chromosome location. Horizontal lines indicate chromosome boundaries. Positive and negative values indicate frequencies of tumors showing copy number increase and decrease, respectively, with gains and losses as described in the method section.

By using two different threshold values (log_2 _ratio >|0.5|, and |1|), we distinguished low-level CNAs, which can result from aneuploidy, from high-level CNAs, which result from focal amplification or deletions of chromosome regions. Using the GISTIC algorithm, the 17q12-q21-amplicon exhibited the highest score index because it was the most frequently amplified with the highest level of amplification. In agreement with previous reports [1,37], the frequency plot analysis showed additional regions of recurrent gene copy number gains and amplification including 1q22-24, 8p12, 8q21-24, 11q13, 17q, 20q13.3 regions (Figure 1).

The histoclinical features and molecular subtypes of the 54 *ERBB2*-amplified BCs are listed in Additionnal file 1-Table S1. They contained 20 IBC and 34 NIBC as well as 10 and 21 primary breast tumors of young (≤ 35 years) and older women (≥ 45 years) (YWBC and OWBC, respectively). The presence of IBC samples among the 54 *ERBB2*-amplified tumors reflects the fact that IBCs do comprise a large proportion of ERBB2 cancers. The *ERBB2*-amplified cases were mainly ductal tumors with pathological grade 3, high expression of ERBB2, TP53 and high Ki67 proliferative index. Moreover, 53% and 6% of the *ERBB2*-amplified BCs were of ERBB2-like and luminal B subtypes, respectively.

### Heterogeneity of *ERBB2*-amplified tumors

We first established the unsupervised hierarchical clustering of the 54 samples and the 225,388 aCGH oligonucleotide probes (excluding X and Y chromosomes) (Figure 1). The samples were separated in two groups (I and II) of 26 and 28 samples, respectively. Histoclinical features were compared between the two groups except for TP53 and Ki67 status because of a too low number of informed samples. For the other histoclinical features, no difference was observed between the two groups except for ER expression (p < 0.05) (Additionnal file 1-Table S3). These results showed heterogeneity of *ERBB2*-amplified tumors with at least two categories, ER- and ER+.

ER and PR status were not available for seven samples (Figure 1). To evaluate the robustness of the clusters, we established the hierarchical clustering without these seven samples (data not shown) and compared it with the clustering analysis including them. The results showed a similar distribution (accuracy = 98%) and confirmed that ER+ and ER- *ERBB2*-amplified breast cancers are different (Fisher exact's test, p = 0.019; O.R. = 4.39).

**Figure 2:**
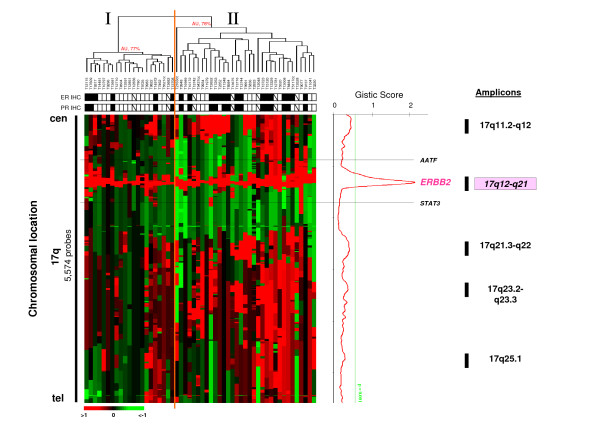
**17q12-q21-amplicon as the most significant on 17q**. Unsupervised hierarchical clustering of genome copy number profiles measured for 54 *ERBB2*-aplified primary breast tumors by aCGH on 5,574 17q probes. Legend is similar to Figure 1. The bar to the left indicates chromosome 17q location with centromere to the top and qter to the bottom. Below the dendrogram, name of tumors are given and the rows indicate their ER and PR IHC status (black square, ER+ or PR+; white square, ER- or PR-). Color codes and corresponding legends are indicated in the box located to the top right. On the right, combining the CNA frequency and gene amplification level, the GISTIC algorithm plotted the score index as a function of chromosome location. *AATF *and *STAT3 *delineate the genomic fragment that defined the *ERBB2-*amplicon. The green line indicates the threshold of significance for the score. Previous studies have reported that the 17q arm is the site of multiple amplicons. They are indicated by black bars at the right of the plot score. The Figure shows only 17q12-q21 (pink box) centered on the *ERBB2 *locus as the significant 17q amplicon (p < 0.001).

We focused on the profiles of chromosome 17 long arm (Figure 2). Unsupervised hierarchical clustering of the 54 samples and the 5,574 oligonucleotide probes covering 17q (Figure 2) separated the samples in two groups (I and II) of 28 and 26 samples, not different with respect to histoclinical features. This separation was influenced by the amplicon size and the gene copy number associated with the multiple amplifications on 17q. The GISTIC score based on the calculation combining CNA frequency and gene amplification level showed that only the 17q12-q21 (pink box) amplicon centered on the *ERBB2 *locus was significant (p < 0.001) (Figure 2).

**Figure 3:**
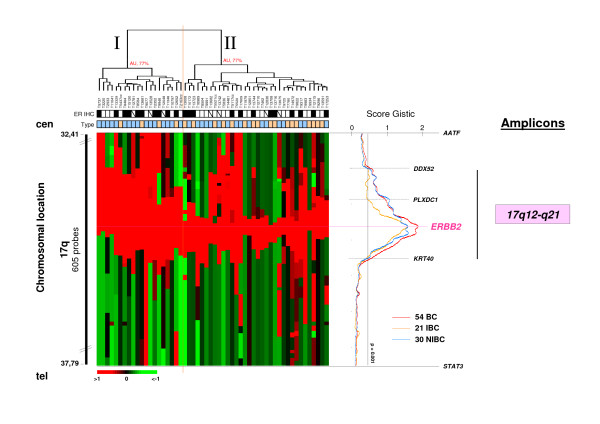
***ERBB2-*amplicon size is significantly different in *ERBB2*-amplified IBC and NIBC**. Unsupervised hierarchical clustering of genome copy number profiles measured for 54 *ERBB2*-amplified primary breast tumors by aCGH on 650 17q12-q21 probes within the genomic interval defined from centromere to telomere by [*AATF*-*STAT3*] (bar to the left) (Additionnal file 1-Table S5). Below the dendrogram, name of tumors are given and the row indicates their ER (black square, ER+; white square, ER-) and IBC/NIBC (orange and blue boxes, respectively) status, respectively. Corresponding legends are indicated in the box located to the top right. On the right, the scores obtained for all BCs, IBCs and NIBCs are plotted (red, orange and blue lines, respectively) as a function of chromosome location. *ERBB2-amplicon *size varies within a region delimited by *DDX52 *and *KRT40 *genes. IBCs have a smaller amplicon than NIBCs located within a region delimited by *PLXDC1 *and *KRT40 *genes.

By next focusing on a smaller interval comprised between *AATF *and *STAT3 *genes (Figure 3), we found that the *ERBB2*-amplicon size varied within a region delimited by *DDX52 *and *KRT40 *genes from centromere to telomere, respectively (Figure 3). Hierarchical clustering of the 54 samples and the 650 oligonucleotide probes covering this region separated the samples in two groups (I and II) of 20 and 34 samples, respectively. The *ERBB2*-amplicon size was larger in group I than in group II. As shown in Figure 3, IBCs had a smaller amplicon than NIBCs, and hormonal receptor negative tumors (ER- and PR-) a smaller amplicon than hormonal receptor positive tumors (ER+ and/or PR+) (p = 0.04 and p = 0.03, respectively) (Additionnal file 1-Table S4). Group II contained twice more IBC tumors than group I. IBCs have a poorer prognosis than NIBCs. The absence of 5-year MFS difference between the two groups suggested that the presence of the *ERBB2*-amplicon influences the NIBC phenotype and generates in NIBCs the same pejorative evolution as that observed in IBCs.

Taken together, our results identified two types of heterogeneity within *ERBB2*-amplified tumors, i.e. with respect to ER status and to IBC/NIBC clinical forms.

To evaluate the potential impact of the ER status on the amplicon size changes linked to IBC and NIBCs, we repeated the analysis using ER- and and ER+ grade-matched IBC and NIBC samples. The number of grade 1 and 2 tumors ER- and ER+ IBC and NIBC samples was too low to be compared in such analysis. Therefore, 21 samples of grade 3 ER- IBC (N = 10) and NIBC (N = 11) were compared (data not shown). The results showed that grade 3 ER- IBCs have a smaller *ERBB2*-amplicon than grade 3 ER- NIBCs. A similar result was obtained with grade 3 ER+ IBC (N = 7) and NIBC (N = 6) samples (data not shown). These results suggest that the size of the ERBB2 amplicon is smaller in IBC than in NIBC and that this is independent of the ER status.

### Size, core and magnitude of the *ERBB2*-amplicon

To better define the *ERBB2-*amplicon, we extracted the data associated with gene copy number amplification for the 54 BCs and the 14 cell lines within the region comprised between *AATF *and *STAT3 *(Additionnal file 1-Table S4). For each of the 54 samples, we precisely defined centromeric and telomeric borders and the size of the *ERBB2-*amplicon by measuring the log_2 _ratio for each oligonucleotide of the region (Additionnal file 1-Table S5). The amplicon borders were defined as first genes targeted by amplification at the centromeric and telomeric locations of the *ERBB2*-amplicon. *PLXDC1*, *FBXL20*, *MED1*, *CRKRS *and *STARD3 *were identified as frequent centromeric borders and *IKZF3*, *PSMD3*, *THRA, WIPF2*, *CDC6 *and *RARA *as frequent telomeric borders. Overall, *CRKRS *and *IKZF3 *were the most frequent centromeric and telomeric borders, respectively.

The *ERBB2*-*C17orf37*-*GRB7 *genomic segment was systematically amplified in all *ERBB2*-amplified samples and constitutes the core of the amplicon.

Finally, we evaluated the *ERBB2 *copy number averages by aCGH in 22 ER+, 25 ER-, 20 IBC and 31 NIBC cases. They were equal to 11.2, 10.7, 10.5 and 11.8 respectively, indicating that the magnitude of the amplification is not different in the different entities.

### Chromosomal regions altered by CNAs in *ERBB2*-amplified tumors

To identify other regions frequently targeted by CNA in the *ERBB2*-amplified tumors, we used the GISTIC algorithm. In addition to the 17q12-q21 amplicon, the score index pointed to 17 other regions affected by CNAs (p < 0.001) (Additionnal file 1-Table S6A) including six regions of gain (1p36.33-p36.32, 4q13.3, 8q23.3-q24.21, 11q13.5-q14.1, 14q11.1-q11.2, 19q12) and eleven regions of loss (4p16.3, 7p22.3, 8p23.3-p23.2, 8p11.23-p11.22, 9q34.3, 10q26.3, 11p15.5, 14q32.33, 15q11.2, 16p13.3, 19p13.3). Including the 17q12-q21 genes, a total of 282 genes were targeted (Additionnal file 1-Table S6A). Regions 4q13.3, 8q23.3-q24.21, 11q13.5-q14.1, 14q11.1-q11.2, 17q12-q21.2, 19q12 were also amplified.

### Correlation between mRNA expression and CNA in *ERBB2*-amplified tumors

To identify genes whose expression levels were modified in proportion to CNA, we combined aCGH and RNA expression data for 51 samples in integrated analyses, as described [3]. Within altered chromosomal regions, relations between gene expression and CNA level were assessed by Pearson correlation test (p < 0.01) on genes for which variations (at both level, gene CNA and deregulated expression), were explained by more than 10% of tumors. The cut-off of 10% was arbitrary defined to increase the robustness of the candidate gene selection. We then identified 1 and 36 genes whose expression levels were downregulated and upregulated in proportion to DNA copy number losses and gains (or amplification), respectively (Additionnal file 1-Table S6A, genes written in bold) (p < 0.01). They were located in 8p23.3-p23.2 (*C8orf68*), 8q23.3-q24.21 (*C8orf53, MAL2, LOC286052, SQLE, KIAA0196*), 11q13.5-q14.1 (*RSF1, INTS4, KCTD21*), 17q12-q21.2 (*DDX52, MRPL45, SOCS7, ARHGAP23, SNIP, MLLT6, CISD3, PCGF2, PSMB3, PIP4K2B, CCDC49, LASP1, LOC642808, CACNB1, LOC90110, FBXL20, MED1, CRKRS, STARD3, PERLD1, ERBB2, C17orf37, GRB7, RAPGEFL1, WIPF2, CDC6, RARA*), and 19q12 (*CCNE1*) regions, respectively. Some genes with correlation between CNA and mRNA expression (Additionnal file 1-Table S6A) have been found in previous studies [1,38].

In the 17q12-q21.2 amplicon, which exhibited the highest frequency (Additionnal file 1-Tables S6A-S6B), amplifications combined to the upregulated gene expression of *CRKRS *(71%), *STARD3 *(82%), *PERLD1 *(86%), *ERBB2 *(92%), *C17orf37 *(84%) and *GRB7 *(86%) were the most frequent (Additionnal file 1-Tables S6A-S6B). This high correlation between gene amplification and gene expression is in agreement with a previous report [39].

### Integrated genome analysis of ER- and ER+ *ERBB2*-amplified tumors

Supervised genome profile analysis identified 43 altered (gains and losses defined with a threshold value of log_2 _ratio >|0.5|) genes with a frequency different in ER- and ER+ *ERBB2*-amplified BCs (Fisher exact test p ≤ 0.05 and False Discovery-Rate (FDR) corrected, Benjamini and Hochberg FDR inferior to 25%) (Additionnal file 1-Table S7A and Additionnal file 2-). Unfortunately, these 43 altered genes could not be validated in a separate cohort of BCs because no high resolution aCGH data (≥ 20,000 oligos) including ER information were available among public data.

Gains targeting *GDPD4, PAK1, CLNS1A *genes at 11q13.5 were associated with ER- *ERBB2*-amplified BCs (Table 1). In ER+ *ERBB2*-amplified BCs, 40 genes were altered by (i) copy number gain at 8p11.23 and 8q23.3-q24.21 (27 genes), (ii) amplification at 8q24.13 and 17q12-q21.1 (5 genes), (iii) both gain and amplification at 8q24.13 (2 genes), and (iv) losses or deletions at 1p31.33, 1p36.32, 4p16.3 and 11p15.5 (6 genes) (Table 1). Only genes (N = 17) targeted by CNA in ER+ *ERBB2 *amplified BCs were sites of genomic variation (12 by copy number variation [CNV], 4 by insertion or deletion [InDel] and 1 by inversion), respectively (Table 1).

**Table 1:** Genes altered by CNA with a frequency significantly different in ER+ and ER- *ERBB2*-amplified tumors.

ER status of *ERBB2*-amplified tumors	Targeted by gene CNA	Altered genes	Location	*CNV, Type#*
**ER-**	**gain**	***GDPD4***	11q13.5	
		***PAK1***		
		***CLNS1A***		

**ER+**	**gain**	***ADAM5P***	8p11.23	***Variation_1197, CNV***
		***ADAM3A***		***Variation_1779, CNV***
		TRPS1*	8q23.3	***Variation_42116, InDel***
		***BC040336***		
		***EIF3H***		
		***AL713790***	8q24.11	
		***SLC30A8***		
		***MED30***		
		***SAMD12***	8q24.12	***Variation_3145, CNV***
		***FAM91A1***	8q24.13	
		***C8orf54***		
		***C8orf78***		
		***BC015129***		
		***TMEM65***		***Variation_52365, CNV***
		***CR933665***		
		***RNF139***		***Variation_28765, InDel***
		***TATDN1***		
		***MTSS1***		Variation_43549, CNV
		***LOC157381***		
		***SQLE***		***Variation_34021, InDel***
		***KIAA0196***		
		***NSMCE2***		***Variation_41128, Indel***
		***TRIB1***		
		***BC038572***	8q24.21	
		***FAM84B***		***Variation_37296, Inversion***
		PVT1*		***Variation_8615, CNV***
		***BC009730***		
	**amplification**	***C8ORFK23***	8q24.13	
		***LOC650095***		
		***LOC90110***	17q12	***Variation_5006, CNV***
		***FBXL20***		***CNVVariation_5006, CNV***
		***CASC3***	17q21.1	
	**gain or amplification**	***ZNF572***	8q24.13	
		***AK093407***		
	**loss or deletion**	***SAMD11***	1p36.33	***Variation_30362, CNV***
		***AGRN***		***Variation_2294, CNV***
		***GNB1***		***Variation_3276, CNV***
		***AK128532***	1p36.32	
		***RNF212***	4p16.3	***Variation_30190, CNV***
		***B4GALNT4***	11p15.5	***Variation_29880, CNV***

*TRPS1 and *PVT1 *gene expression was in proportion to their CNA

***# ***The information about the presence of CNV and the type of genomic variations (CNV, InDel, inversion...) was extracted from hg17 and hg18.

When aCGH and RNA data were combined (Additionnal file 1-Table S7A), *TRPS1 *and *PVT1 *were found associated with ER+ *ERBB2*-amplified tumors (Table 1).

### Gene expression and canonical pathways of ER- and ER+ *ERBB2*-amplified tumors

We established the gene expression profiles of 51 of the 54 samples. Hierarchical clustering did not distinguish ER- *ERBB2*-amplified BCs from ER+ *ERBB2*-amplified BCs (Additionnal file 4-Figure S2). The 51 BCs were separated in two groups (I and II) of 25 and 26 cases, respectively. No difference in ER protein expression (as defined by IHC) was observed between the two groups (p = 0.25) (Additionnal file 1-Table S7B). Group II contained more IGF1R-expressing tumors (as defined by IHC) and normal-like tumors than group I.

**Figure 4:**
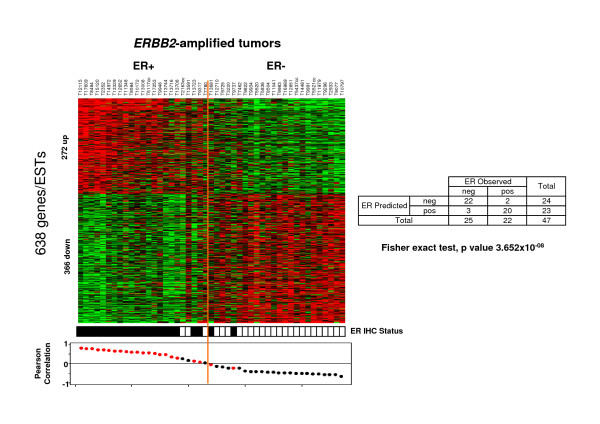
**Supervised classification of ER+ and ER- *ERBB2*-amplified BCs based on RNA expression data**. Left, classification of 51 samples using a 638-gene expression signature (listed in Additionnal file 1-Table S7C). Top panel, matrix of expression data. Each row of the data matrix represents a gene and each column represents a sample. Expression levels are depicted according to the color scale shown at the bottom. Red and green indicate expression levels respectively above and below the median. The magnitude of deviation from the median is represented by the color saturation. Genes are ordered from top to bottom by their decreasing signal-to-noise ratio. Tumor samples are ordered from left to right according to the decreasing correlation coefficient of their expression profile with the median profile of the ER+ samples (Bottom panel). The orange line indicates the threshold 0 that separates the two predicted classes of samples, "ER+ class" (to the left) and "ER- class" (to the right). The middle panel indicates the observed ER status of tumors (black square, ER+; white square, ER-). Right, correlation between the molecular grouping based on the combined expression of the 638 genes (predicted status) and the observed ER status of samples.

Supervised analysis of the 51 samples showed that 402 genes (corresponding to 638 probe sets) were differentially expressed in ER- and ER+ *ERBB2*-amplified tumors (SNR analysis with FDR <0.1%) (Figure 4). They included 257 genes upregulated in ER- *ERBB2*-amplified tumors and 145 upregulated in ER+ *ERBB2*-amplified tumors (corresponding to 366 and 272 probe sets, respectively) (Additionnal file 1-Table S7C). *GATA3*, *ESR1*, *TFF1*, *TFF3 *and *ERBB4 *were upregulated, whereas *IGF2R*, *GATA6*, *EGFR *and *TGFA *were downregulated in ER+ *ERBB2*-amplified tumors.

Using the Ingenuity software, canonical pathways were defined as associated with either ER- or ER+ *ERBB2*-amplified tumors (p < 0.05) (Additionnal file 1-Table S7D and Additionnal file 5-Figure S3). Genes coding for proteins involved in WNT/β-catenin signaling pathway, aryl hydrocarbon receptor signaling (cell cycle and apoptosis or nuclear receptor signaling), LPS/IL-1 mediated inhibition of RXR function (nuclear receptor signaling) as well as various metabolic pathways (i.e. Nicotinate and nicotinamide, galactose, Inositol Phosphate, β-Alanine) were upregulated in ER- *ERBB2*-amplified tumors (p < 0.05). In contrast, among genes downregulated in ER- *ERBB2*-amplified tumors (p < 0.05), some coded for proteins involved in endothelin-1 signaling, nitric oxide signaling in the cardiovascular system (both associated with cardiovascular signaling pathways), NRF2-mediated oxidative stress response (cell stress and injury pathway), synaptic long term depression, neuropathic pain signaling in dorsal horns neurons, mechanisms of viral exit from host cells and other metabolic pathways.

### Chromosomal regions altered by CNAs in NIBC and IBC *ERBB2*-amplified tumors

To identify regions frequently targeted by CNA in IBC and NIBC *ERBB2*-amplified tumors, we used the GISTIC algorithm. The score index pointed to 4 gained or amplified regions (8q23.3, 17q11.1-q11.2, 17q12-q21.2 and 17q21.32-q21.33) in NIBCs and 7 gained or amplified regions (8q24.11-q24.12, 8q24.13, 8q24.13-q24.21, 17q11.2, 17q12-q21.2, 17q21.33 and 19q12) in IBCs (p < 0.001) (Additionnal file 1-Table S7E). A total number of 172 and 113 genes were targeted in NIBC and IBC populations, respectively. Among them, 50 genes were commonly found altered (in bold in Additionnal file 1-Table S7E) in the two populations. The number of genes associated with the 17q12-q21 amplicon was lower in IBC than in NIBC in agreement with the size difference of the *ERBB2*-amplicon between the two populations.

### Gene expression differences between IBC and NIBC *ERBB2*-amplified tumors

We compared the expression of genes targeted by the 17q12-q21-amplicon in IBCs and NIBCs. *SNIP *(p = 0.0002), *MLLT6 *(p = 0.0072), *CISD3 *(p = 0.0111), *PCGF2 *(p = 0.0067), *PSMB3 *(p = 0.0029), *PIP4K2B *(p = 0.0126), *FBXL20 *(p = 0.0324), *STARD3 *(p = 0.0318), *GRB7 *(p = 0.0244) and *RARA *(p = 0.0201) were downregulated in *ERBB2*-amplified IBCs compared to *ERBB2*-amplified NIBCs. *SQLE *(8q24.13) (p = 0.0268) and *RSF1 *(11q13.5) (p = 0.0067) were upregulated in *ERBB2*-amplified IBCs.

To better understand the mechanisms associated with gene expression deregulation of these candidates in IBC and NIBC we looked in detail at their genomic alterations. Among the 10 genes downregulated in *ERBB2*-amplified IBCs compared to *ERBB2*-amplified NIBCs, we noted that *SNIP *and *RARA *were gained or amplified in NIBCs but not in IBCs. *MLLT6, CISD3, PCGF2, PSMB3, PIP4K2B, FBXL20, STARD3, GRB7 *were gained or amplified in both populations. This suggests that downregulation of these 10 genes in IBC could be driven by epigenetic mechanisms differentially regulated in the two populations. Among the 2 genes upregulated in *ERBB2*-amplified IBCs compared to *ERBB2*-amplified NIBCs, *SQLE *(8q24.13) was gained or amplified in IBCs but not in NIBCs while *RSF1 *(11q13.5) was not targeted by CNA in the two populations. This suggests that the upregulation of *SQLE *associated with gain or amplification could be a specific oncogenic mechanism in IBC whereas the upregulation of *RSF1*could be explained by others mechanisms such as hypomethylation of its promoter.

### *ERBB2 *amplification, gene expression, protein expression and phsophorylation

**Figure 5:**
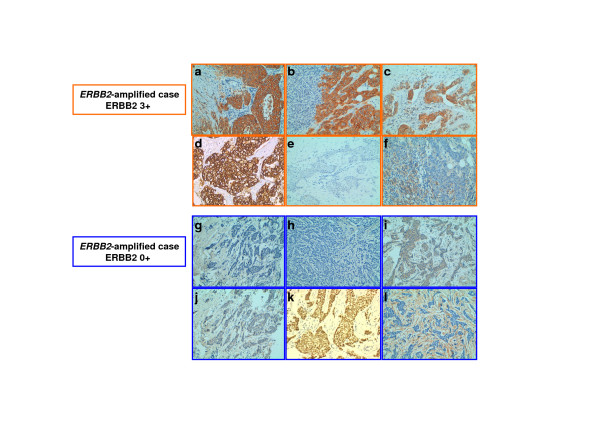
**Immunohistochemical expression analysis in *ERBB2*-amplified tumors**. Two exemples of IHC profile of *ERBB2-*amplified cases. The IHC profiles show in *ERBB*2-amplified ERBB2-positive (a-f) an ERBB2 expression defined as 3+ with Herceptest (a) and TAB250 (b) as well as a cytoplasmic positivity of pERBB2 (c) a cell membrane positivity of IFG1R (d) and an absence of FOXA1 (e) and EGFR (f) expression. The IHC profiles show in *ERBB*2-amplified ERBB2-negative (g-m) an ERBB2 expression defined as 0+ with Herceptest (h) and TAB250 (i) as well as a weak cytoplasmic positivity of pERBB2 (j) no positivity of cell membrane of IFG1R (k), a strong nuclear expression of FOXA1 (l) and an absence of EGFR (m).

ERBB2 status was assessed by IHC. First, we used the HerceptTest and the TAB250 antibodies to evaluate the ERBB2 expression but also to compare their detection in relation with the *ERBB2*-amplification. Therefore, using HerceptTest on 40 *ERBB2*-amplified BCs (Figure 5), 97.5% (39/40) of the samples were ERBB2-positive (Additionnal file 1-Tables S7-S8A). They were classified 2+ (1 ERBB2-like, 1 luminal A, 2 basal and 1 normal-like) and 3+ (15 ERBB2-like, 2 luminal A, 2 luminal B, 2 basal, 6 normal-like, 7 not assigned). Only one (luminal A) was 0+ (Additionnal file 1-Table S8). TAB250 on 35 *ERBB2*-amplified BCs detected 23 positive samples (66%) (Additionnal file 1-Tables S8-S9A). A total of 35 tumors were analyzed with both HercepTest and TAB250. Among the 23 samples positive for TAB250, all (100%) were also HerceptTest positive (Additionnal file 1-Table S8). Conversely, among the 12 tumors negative for TAB250, only one was HerceptTest negative (Additionnal file 1-Table S8).

*ERBB2 *copy number averages evaluated by aCGH in tumors positive with HerceptTest (N = 39), TAB250 (N = 23) or both were equal to 11.3, 13.3 and 13.3 respectively (Additionnal file 1-Table S8). Tumors negative for HerceptTest (N = 1), TAB250 (N = 12) or both were equal to 8.8, 7.8 and 8.8, respectively. Among samples positive with both HerceptTest and TAB250, 41% (16/39) and 48% (11/23) were subtyped as ERBB2-like, respectively. Similarly, among samples positive with both HerceptTest and TAB250, 5% (2/39) and 4% (1/23) were subtyped as luminal B, respectively. Taken together, these results suggest a linear relationship between *ERBB2 *gene amplification and its gene and protein expressions for almost half of *ERBB2*-amplified samples. This relationship was not perfect for 4/12 amplified samples negative with TAB250 were ERBB2-like, respectively. However, we noted that they were all 3+ with HerceptTest. The only *ERBB2*-amplified sample negative with HerceptTest (T11348) was luminal A. Unfortunately, no sufficient material (T11348) associated with a correct morphology was available to confirm its *ERBB2 *amplification status by using silver in situ hybridization (SISH, Ventana). The IHC pattern of this sample showing also FOXA1 positivity was in agreement with its luminal A phenotype [40,41], suggesting that no mismatching had occurred for this case.

The expression of pERBB2 could help predict clinical response of ERBB2-positive BCs to trastuzumab or lapatinib [8,42,43]. pERBB2 status was evaluated by IHC on 22 *ERBB2*-amplified samples (Figure 5) and was detected in 17/22 (Additionnal file 1-Tables S8-S9A). Among the 17 samples, 17 (100%) and 14 (82%) were also HerceptTest and TAB250 positive, respectively. This result suggests a good correlation between ERBB2 expression and phosphorylation status (Table 2).

**Table 2:** Co-expression frequency and association between protein expressions

Protein combinations	Co-expression*
	N (%)
pERBB2/ERBB2	81% (17/21)
IGF1R/ERBB2	24% (7/29)
IGF1R/pERBB2	14% (2/14)
EGFR/ERBB2	33% (12/36)
EGFR/pERBB2	41% (7/17)
IGF1R/EGFR	11% (1/9)
IGF1R and EGFR/ERRB2	3% (1/29)
IGF1R and EGFR/pERBB2	0% (0/14)
FOXA1/ERBB2	67% (14/21)
FOXA1/pERBB2	71% (12/17)

*Co-expression means that tumors were positive for both factors. ^†^Expression scores evaluated according to scales defined in material and methods to examine correlations.

### IGF1R and EGFR expression in *ERBB2*-amplified tumors

Expression levels of insulin-like growth factor type 1 receptor (IGF1R), epidermal growth factor receptor (EGFR), and ERBB2 have been linked to clinical outcome in breast and other solid tumors [44]. We extended our IHC analysis to define a potential impact of IGF1R and EGFR protein expression in *ERBB2*-amplified patients.

IGF1R status was assessed in 30 *ERBB2*-amplified BCs for which ERBB2 IHC status had been determined (Figure 5). Seven (23%) tumors expressed IGF1R (Additionnal file 1-Tables S8-S9A); all of them also expressed ERBB2 (all HerceptTest 3+). Similarly, EGFR status was assessed in 37 *ERBB2*-amplified BCs for which ERBB2 status was established. Twelve (32%) tumors expressed EGFR (Additionnal file 1-Tables S8-S9A); all of them expressed ERBB2 (HerceptTest: 9 3+ and 3 2+). IGF1R/EGFR/ERBB2 coexpression was rarely observed (1/29) while IGF1R/ERBB2 and EGFR/ERBB2 coexpression was observed in 24% (7/29) and 33% (12/36) of cases, respectively (Table 2). IGF1R/EGFR/pERBB2 coexpression was never observed (0/24) while IGF1R/pERBB2 and EGFR/pERBB2 coexpression was observed in 14% (2/14) and 41% (7/17), respectively (Table 2). These results are in agreement with the signaling cross-talk described between EGFR family members and IGF1R in maintaining the malignant phenotype [45].

### FOXA1 is frequently coexpressed with ERBB2 in *ERBB2*-amplified tumors

Forkhead transcription factor FOXA1 is involved in mammary tumorigenesis and may be involved in a cross-talk between hormonal receptors and ERBB2 [41]. To define a potential impact of FOXA1 expression in *ERBB2*-amplified BCs, FOXA1 status was assessed by IHC in 22 *ERBB2*-amplified BCs for which ERBB2 IHC status had been determined (Figure 5). Fifteen (68%) tumors expressed FOXA1 (Additionnal file 1-Tables S8-S9A). FOXA1 expression was observed in 67% (14/21) of ERBB2-positive tumors. In details, 1 (luminal A) and 14 tumors (1 luminal B, 9 ERBB2-like, 3 normal-like and 1 not assigned) were classified as 0+ and 3+, respectively. This result is in agreement with a recent study showing that FOXA1 was expressed in all ERBB2-positive breast cancer cell lines [46]. FOXA1 expression was observed in 71% (12/17) of pERBB2-tumors (Table 2).

### Clinical features and protein expression analysis of ER+ and ER- *ERBB2*-amplified BCs

We finally compared clinical features and EGFR, FOXA1, ERBB2, pERBB2 and IGF1R protein expression in ER+ (N = 22) and ER- (N = 25) *ERBB2*-amplified tumors (Additionnal file 1-Table S9B). Compared to ER+ cases, ER- cases were all PR-negative (p < 0.001), more often EGFR-positive (p < 0.05) and ERBB2-like. ER- BCs had a tendency (higher grade and 5 years-MFS [p = 0.059 and p = 0.06, respectively]) to have a poorer prognosis and were more likely to recur than ER+ BCs (Additionnal file 1-Table S9B).

## Discussion

We used high-resolution aCGH and DNA microarrays to define the genome and gene expression profiles of BCs with 17q12-q21-amplification. Results concerning cell lines are given for information to the scientific community.

At this stage, we would not discuss any relation between *ERBB2 *amplicon size and resistance to trastuzumab because of small numbers.

### Genomic alterations of *ERBB2*-amplified tumors

We identified 18 regions targeted by CNAs in ERBB2-amplified BCs. As expected, the most frequent encompassed the region of *ERBB2*-amplification previously defined [17,37] and included *NEUROD2, PPP1R1B*, *STARD3*, *TCAP*, *PNMT*, *PERLD1*, *ERBB2*, *C17ORF37*, *GRB7 *and *IKZF3*/*ZNFN1A3 *17q12 genes (>85% of 17q12-q21-amplified samples) (Additionnal file 1-Table S5). *ERBB2*-*C17orf37*-*GRB7 *was the core region systematically included in all 17q12-q21-amplified samples.

We noted that 8q24.1, 11q13.4-q14.1, 14q11.1-q11.2, 17q21.1-q21.2 and 19q12 regions of amplification were associated with *ERBB2*-amplified BCs. Some of these regions were previously reported co-amplified in breast cancers [1] (Additionnal file 1-Table S6A). A recent study [47] showed correlations between the presence of such amplicons and molecular subtypes in grade 3 invasive ductal BCs (Additionnal file 1-Table S6A) suggesting that (i) the two amplified subregions within 8q24.1 amplifications (with a frequency of 50% and 21%) coud be associated with basal and non-basal subtypes, respectively; (ii) the 11q13.4-q14.1 amplification (with a frequency of 15%) could be associated exclusively with the luminal subtype; (iii) the 14q12, 14q12-q13.1 and 14q23.2-q23.3 amplifications (with an identical frequency of 12%) coud be associated exclusively with the ERBB2 subtype; (iv) the 17q21.1-q21.2 amplification (with a frequency of 100%) coud be associated exclusively with the ERBB2 subtype. In contrast, for other 17q amplicons (17q21.32, 17q21.32-q22, 17q23.1q23.2 and 17q23.3) we did not found any association with *ERBB2*-amplified tumors; (v) the two amplified subregions within 19q12 amplifications (with frequencies of 12% and 16%, respectively) coud be associated exclusively with the basal subtype.

We showed a genomic heterogeneity of *ERBB2*-amplified BCs with respect to with ER+ and ER- status. The genomic profiles of ER+ and ER- *ERBB2*-amplified tumors were previously reported [18]. Gains of 17q23-q24 and losses of 1p39, 1p36, 1p35, 1p32, 7q21-q22, 7q34, 7q36.1-q36.3, 9p21.3, and 11q13.5 were more frequent in ER+ cases while gain of 5p15-p12 was associated with ER- cancers. None of these regions was observed similarly altered in our study (Additionnal file 1-Table S6A).

We identified 37 genes whose expression levels were deregulated in proportion to CNA. Some of these genes were similarly found in previous studies [1,48] (*SQLE, KIAA0196*, *STARD3, PERLD1, ERBB2, GRB7, CCNE1*). In ER+ *ERBB2*-amplified BCs, only *PVT1 *was commonly found with this study [1]. Similarly, 24 genes (65%) from another previous study [38] were common with our candidates (Additionnal file 1-Table S6A). Seventeen (*SQLE, DDX52, MRPL45, SOCS7, PSMB3, PIP4K2B, CCDC49, LASP1, FBXL20, MED1, STARD3, PERLD1, ERBB2, C17orf37, GRB7, RAPGEFL1*, and *CCNE1*) and three (*KIAA0196, RSF1, INTS4*) were associated with ERBB2 and luminal molecular subtypes, respectively; *KCTD21 *and *PERLD1 *were associated with both ERBB2 and luminal molecular subtypes. *MAL2, WIPF2 *and *CDC6 *were not associated with any specific molecular subtype [38].

The difference in the number of genes found in our study compared to previous studies [1,38,47,48] could be explained by the high resolution aCGH 244K we used (whereas 2464 BAC arrays [OncoBAC array] and 32K BAC arrays were previously used [[1] and [38,47,48], respectively]) and by integrating gene level with gene expression analysis.

Within the 17q12-q21 region, amplification of *ERBB2*, *STARD3*, *TCAP*, *PNMT*, *PERLD1*, *C17orf37, GRB7*, *GSDML*, *PSMD3 *and *THRAP4 *genes have been reported to correlate with gene expression [1,15,28,36]. Amplification and overexpression of other genes (such as *GRB7, C17orf37 *and *STARD3*) of the 17q12 region could contribute to tumor growth [18,19]. *C17orf37 *open reading frame encodes a 12-kDa protein of unknown function. C17ORF37 protein expression is linked with *ERBB2*-amplification in most cases, however, it has also been observed in breast carcinomas that do not overexpress ERBB2, and particularly in early stage and infiltrating lobular carcinomas that typically do not overexpress ERBB2 [49]. This suggests that C17ORF37 could represent an additional target for cancer therapy. Growth factor receptor-bound protein 7 (GRB7) is an adaptor-type signaling protein that binds to a variety of cell surface receptor tyrosine kinases including EGFR and ERBB2 [50] to mediate downstream signaling pathways. GRB7 may facilitate ERBB2-mediated signal transduction and tumor formation [51] and has been suggested as a therapeutic target [19].

Seventeen other regions were affected by CNAs with a lower frequency in *ERBB2*-amplified tumors. Some of them have been found involved in mammary carcinogenesis. Loss of 4p16.3 (Additionnal file 1-Table S6A) was telomeric to the deleted R4 region located between D4S43 and D4S127 (4p16.3) previously observed in breast carcinomas [52]. It included *CTBP1*, whose decreased expression has been associated with migratory, invasive potential of melanoma cells [53]. However, we did not observe downregulated *CTBP1 *expression in proportion to the deletion of this region. The 4q13.3 gain/amplification targeted 20 genes, including those encoding EGFR ligands *EPEG*, *EREG*, *AREG *and *BTC*, which could play a critical role in oncogenesis [54]. Loss of 8p23.3, a region affected by deletion in both breast and pancreatic cell lines [55] targeted 10 genes including *ARHGEF10*, a potential tumor suppressor [55]. However, this gene did not show downregulated expression in proportion to copy number loss.

Gain and amplification of 8q23.3-q24.21 targeted 31 genes. This region delimited from centromere to telomere by *TRPS1 *and *PVT1 *contains *MYC*, two colorectal cancer risk loci, rs16892766 (8q23.3) [56] and rs6983267 (8q24.21), and one breast cancer risk locus rs13281615 (8q24.21) [57]. rs6983267 is a good candidate for a multicancer susceptibility marker [58]. A strong association for rs13281615 was observed for ER+, PR+, and low grade breast tumors [57]. To date, no relationship has been reported between the presence of these loci and the 8q23.3-q24.21 amplification. Coamplification of *MYC *and *PVT1 *seem to correlate with rapidly growing and progressive breast cancer and has been associated with poor outcome in postmenopausal or ERBB2-positive BC patients [59]. Only *C8orf53, MAL2, LOC286052, SQLE *and *KIAA0196 *were upregulated in proportion to copy number gain or amplification of the 8q23.3-q24.21 region in the *ERBB2*-amplified tumors. The *MAL2 *gene was previously found amplified and overexpressed in breast and other cancers, yet the significance of this is unknown. *SQLE *overexpression was found in high-risk ER+ stage I/II BCs [60] but *SQLE *mRNA overexpression was not different in ER+ and ER- *ERBB2*-amplified tumors.

Amplification of 8q24.11-13 (*THRAP6, DCC1, SQLE, SPG8*) and 11q14.1 (*NDUFC2, ALG8, USP35*) have been associated with poor prognosis in a novel subtype of high-grade ER- tumors [36]. Within 11q13.5-q14.1, *RSF1, INTS4 *and *KCTD21 *were upregulated in proportion to copy number gain/amplification in the *ERBB2*-amplified tumors. *RSF1*, a chromatin-remodeling gene, was identified as a potential oncogene in ovarian serous carcinoma [61].

Within 19q12, *CCNE1 *was upregulated in proportion to copy number gain or amplification. CCNE1 expression in breast cancer cells has been associated with ER- status, ERBB2 expression, high tumor grade and high proliferation index [62]. Breast cancer-associated variants have been found in four cell cycle genes including *CCNE1 *rs997669 [63].

### ER- and ER + *ERBB2*-amplified breast tumors

Expression profiling studies [11,64] have also suggested that *ERBB2*-amplified BCs constitute a heterogeneous group that could be subdivided according to ER status: ER+ *ERBB2*-amplified BCs fall into the luminal B cluster; and ER- *ERBB2*-amplified BCs constitute the actual ERBB2-like subtype.

Specific genetic aberrations and expression are indeed characteristic of ER+ and ER- ERBB2 BCs. Upregulated *ESR1*, *GATA3*, *ERBB4, TFF1 and TFF3 *gene expression was associated with ER+ *ERBB2*-amplified tumors. These genes are typically associated with the luminal subtype, suggesting that the ER+ *ERBB2*-amplified tumors could be a branch of the luminal tumors and could share the same progenitor. Only *TRPS1 *and *PVT1 *were identified as candidate oncogenes in ER+ *ERBB2*-amplified tumors. *TRPS1 *encodes a zinc finger transcription factor widely expressed in human tissues and overexpressed in BCs [65]. *PVT1 *is most likely a noncoding RNA that acts independently of *MYC *and, when amplified and overexpressed, increases proliferation and inhibits apoptosis [66]. Seven miRNAs cover *PVT1 *[67] and could play a role in mammary oncogenesis.

The ER- *ERBB2*-amplified samples were mainly ERBB2-like (88%) (Additionnal file 1-Table S9B) and upregulated *IGF2R*, *GATA6*, *TGFA *and *EGFR*. The canonical WNT/β-catenin signaling pathway was associated with ER- *ERBB2*-amplified tumors. β-catenin is a substrate of ERBB2 kinase. Geldanamycin could be included in the panel of potential therapeutic tools because it destabilizes ERBB2 tyrosine kinase and suppresses WNT/β-catenin signaling in ERBB2-overexpressing cells [68].

### IBCs and NIBCs with *ERBB2*-amplification

We found that the size of *ERBB2 *amplicon is smaller in IBC than in NIBC and that this difference is independent of the ER status. Perhaps the rapid evolution of IBC prevents the construction of large amplicons.

Twelve genes were deregulated in *ERBB2*-amplified IBCs. Ten of them (*SNIP, MLLT6, CISD3, PCGF2, PSMB3, PIP4K2B, FBXL20, STARD3, GRB7 *and *RARA*) are located within the 17q12-q21-amplicon suggesting that this region has a strong influence on the IBC phenotype in *ERBB2*-amplified BCs. Some of the 10 downregulated genes are potential tumor suppressor genes. The SNIP protein is tyrosine phosphorylated upon integrin-dependent adhesion or EGF treatment, with a potential role as a downstream effector of cell matrix and growth factor signaling that suppresses tumorigenic properties of breast cancer cells [69]. PCGF2 is a polycomb protein that could act as a tumor suppressor [70].

### Markers and targets in *ERBB2*-amplified BCs

The response of *ERBB2*-amplified BCs to anthracyclines may be influenced by the presence of *TOP2A *in the 17q12-q21-amplicon [20,21,37]. Although *TOP2A *amplification was described as a discriminatory feature between ER+ and ER- *ERBB2*-amplified BCs [48] we were not able to confirm this result because the statistical analysis was not applicable to our data. (Data not shown)

IGF1R+ or EGFR+ *ERBB2*-amplified tumors were all ERBB2+ while IGF1R and EGFR were rarely coexpressed. Previous studies have suggested that interactions among families of growth factor receptors enhance the malignant behavior of tumor cells [71]. Furthermore, cross-talk between IGF1R and EGFR or ERBB2 has been implicated in the development of resistance to EGFR and ERBB2 inhibitors [46]. FOXA1 was frequently coexpressed with ERBB2 in *ERBB2*-amplified tumors. FOXA1 is essential for optimal expression of half of ER-related genes. FOXA1 expression correlates with luminal A subtype and good prognosis [72]. We did not find any association between FOXA1 expression and the outcome of patients with *ERBB2*-amplified tumors.

## Conclusions

Our study shows that ERBB2 BCs are heterogeneous with respect to clinical, immunohistochemical and molecular factors and identifies features that may be useful in the design of therapeutical approaches of these poor prognosis cancers.

## Competing interests

The authors declare that they have no competing interests.

## Authors' contributions

All authors read and approved the final manuscript. FS, IB and JA did the genomic profiling and data analysis. PF, CI, and JB did the transcriptome experiments and data analysis. IB, SR, ABH and JJ did the immunohistochemistry and data analysis. ECJ and JJ are pathologists and were responsible for clinical features collections. PV, CT, FBA and FB are physicians in charge of the patients. FB, DB, and MC did project planning, integrated data analysis and manuscript writing.

## Pre-publication history

The pre-publication history for this paper can be accessed here:

http://www.biomedcentral.com/1471-2407/10/539/prepub

## Supplementary Material

Additionnal file 1 **Supplementary Tables**. Table S1 - Clinical and histological features of the 54 profiled *ERBB2*-amplified breast tumors. Table S2 - Proteins tested by IHC: antibodies and experimental conditions. Table S3 - Clinical and histological features of the two aCGH-clustered *ERBB2*-amplified tumor groups defined through the whole genome. Table S4 - Clinical and histological features of the two aCGH-clustered *ERBB2*-amplified tumor groups defined through the [*AATF-STAT3*] genomic segment. Table S5 - Definition of the *ERBB2 *amplicon score. Table S6A - Significant altered regions found in the 54 samples harboring the 17q12-q21-amplification (defined by the score index with a threshold of 10^-3^). Table S6B - Gene expression deregulation frequencies of genes included in *ERBB2*-amplicon. Table S7A - Integrated genome analysis of ER- and ER+ *ERBB2*-amplified tumors. Table S7B- Clinical and histological features of the two clustered *ERBB2*-amplified tumor groups defined using gene expression data. Table S7C - Genes with expression significantly different in ER- and ER+ *ERBB2*-amplified tumors. Table S7D - Canonical pathways associated with ER+ and ER- expression signature in *ERBB2*-amplified BCs. Table S7E - Regions significantly altered by CNA in *ERBB2*-amplified IBC and NIBC. Table S8 - Transversal analysis of *ERBB2*-amplified BCs. Table S9A - Clinical features and protein expression analysis of ERBB2-amplified BCs. Table S9B - Clinical features and protein expression analysis of ER+ and ER- *ERBB2*-amplified BCs. Click here for file

Additionnal file 2 **Supplementary Material**. Click here for file

Additionnal file 3 **Figure S1: Genomic profiles of chromosome 17 in *ERBB2*-amplified primary breast tumors and breast cancer cell lines**. A-C - Regional 17q12-q21 amplification centered on the *ERBB2 *locus observed in the 54 studied BCs. S1A and S1B-C show genomic profiles of chromosome 17 established with CGH analytics^® ^software (Agilent Technologies) in IBC and NIBC samples, respectively. The 17q12-q21 amplification (log_2 _ratio >1) was found as single abnormality or associated with other various copy number aberrations along chromosome 17. The arrow indicates the 17q12-q21-amplicon centered on the *ERBB2 *locus. **D **- Regional 17q12-q21-amplification centered on the *ERBB2 *locus observed in the 14 studied breast cancer cell lines. Genomic profiles of chromosome 17 were established as defined in Additionnal file 3-Figures S1A-C. Click here for file

Additionnal file 4 **Figure S2: Whole-genome expression profiling of *ERBB2*-amplified BCs**. **A **- Hierarchical clustering of 51 samples and 13,114 genes/ESTs with significant variation in mRNA expression level across the samples. Each row of the data matrix represents a gene and each column represents a sample. Expression levels are depicted according to the color scale shown at the bottom. Red and green indicate expression levels respectively above and below the median. The magnitude of deviation from the median is represented by the color saturation. The dendrogram of samples (above matrixes) represents overall similarities in gene expression profiles and is zoomed in B. **B **- Dendrograms of samples. *Top*, Two large groups of tissue samples (designated I to II) are evidenced by clustering and delimited by the orange solid vertical line (see also Additionnal file 1-Table S7B). Below the dendrogram, Below the dendrogram, from the top to the bottom, name of tumors are given and the two first rows indicate their ER and IGF1R status (black square, ER+ or IGF1R+; white square, ER- or IGF1R-) of patients, while the last row indicates the molecular subtypes. The third row indicates the gene expression molecular subtypes as previously defined [13] using specific colored squares (luminal A: dark blue; luminal B: sky blue; basal: red; ERBB2-like: pink; normal-like: green and not assigned: white). Click here for file

Additionnal file 5 **Figure S3: Canonical pathways associated with ER+ and ER- *ERBB2*-amplified BCs**. Histograms show canonical pathways associated with either ER+ or ER- *ERBB2*-amplified BCs. Established by Ingenuity^® ^pathways analysis software, they show relevant proteins (Additionnal file 1-Table S7D) encoded by genes associated with the ER+/ER- *ERBB2*-amplified BCs molecular signature (Additionnal file 1-Table S7C). Color codes and corresponding legends are indicated in the box located to the left at the top of the figure. Click here for file
