# Supplementary material for: Genome profiling of ERBB2-amplified breast cancers
Source: BMC Cancer. 2010 Oct 8;10:539. doi: 10.1186/1471-2407-10-539 (PMC2958950; doi:10.1186/1471-2407-10-539)
Supplement: Additionnal file 2 — Supplementary Material. [file 1471-2407-10-539-S2.DOC]

**Additionnal file 2-Supplementary material**

**ER+/ER- Analysis:**

aCGH analysis:

Fisher exact test was applied on frequencies of CNA in both populations, taking in account that gains and losses were defined with a threshold value of log_2_ ratio >|0.5|). The p values ≤0.05 with FDR corrected [Benjamini and Hochberg False Discovery-Rate] <25% were retained.

aCGH/Expression analysis:

Fisher exact test was applied on frequencies of association CNA/deregulation in both populations, gain/upregulation and loss/downregulation defined as previously reported [22].

t-test was applied in both ER- and ER+ populations (with a threshold, p≤0.05) and Pearson’s correlation was calculated (with a threshold, p≤0.05).

FDR method was not applied on the two last statistical tests because the number of variables to test was not sufficient.

From 2506 probes exhibiting significant CNA in *ERBB2*-amplified tumors, 229 probes corresponded to 43 genes/EST altered with a frequency significantly different in ER- and ER+ *ERBB2*-amplified tumors (**Additionnal file 1-Table S7A**). For simplicity, **Additionnal file 1-Table S7A** does not contain probes but unique genes.

Therefore, 43 genes were found altered with a frequency significantly different in ER- and ER+ *ERBB2*-amplified tumors (noted ER1 and ER0 in **Additionnal file 1-Table S7A**, respectively). Among them, only 2 genes, *TRPS1* and *PVT1* were identified as potential candidate oncogenes associated with ER+ *ERBB2*-amplified tumors. Data are summarized in **Table 1.**

Expression analysis :

SNR with a threshold 1/10^3^ reported 638 discriminating Affymetrix probe sets corresponding to 402 genes/ESTs (**Additionnal file 1-Table S7C**).

Interestingly, *TRPS1* and *PVT1* identified above as potential candidate oncogenes significantly associated with ER+ *ERBB2*-amplified tumors were also included in the upregulated genes in ER+, (from the highest significance, at the 5^th^ and 36^th^ places, respectively).
