# Supplementary material for: Genome profiling of ERBB2-amplified breast cancers
Source: BMC Cancer. 2010 Oct 8;10:539. doi: 10.1186/1471-2407-10-539 (PMC2958950; doi:10.1186/1471-2407-10-539)
Supplement: Additionnal file 3 — Figure S1: Genomic profiles of chromosome 17 in ERBB2-amplified primary breast tumors and breast cancer cell lines. A-C - Regional 17q12-q21 amplification centered on the ERBB2 locus observed in the 54 studied BCs. S1A and S1B-C show genomic profiles of chromosome 17 established with CGH analytics® software (Agilent Technologies) in IBC and NIBC samples, respectively. The 17q12-q21 amplification (log2 ratio >1) was found as single abnormality or associated with other various copy number aberrations along chromosome 17. The arrow indicates the 17q12-q21-amplicon centered on the ERBB2 locus. D - Regional 17q12-q21-amplification centered on the ERBB2 locus observed in the 14 studied breast cancer cell lines. Genomic profiles of chromosome 17 were established as defined in Additionnal file 3-Figures S1A-C. [file 1471-2407-10-539-S3.PDF]

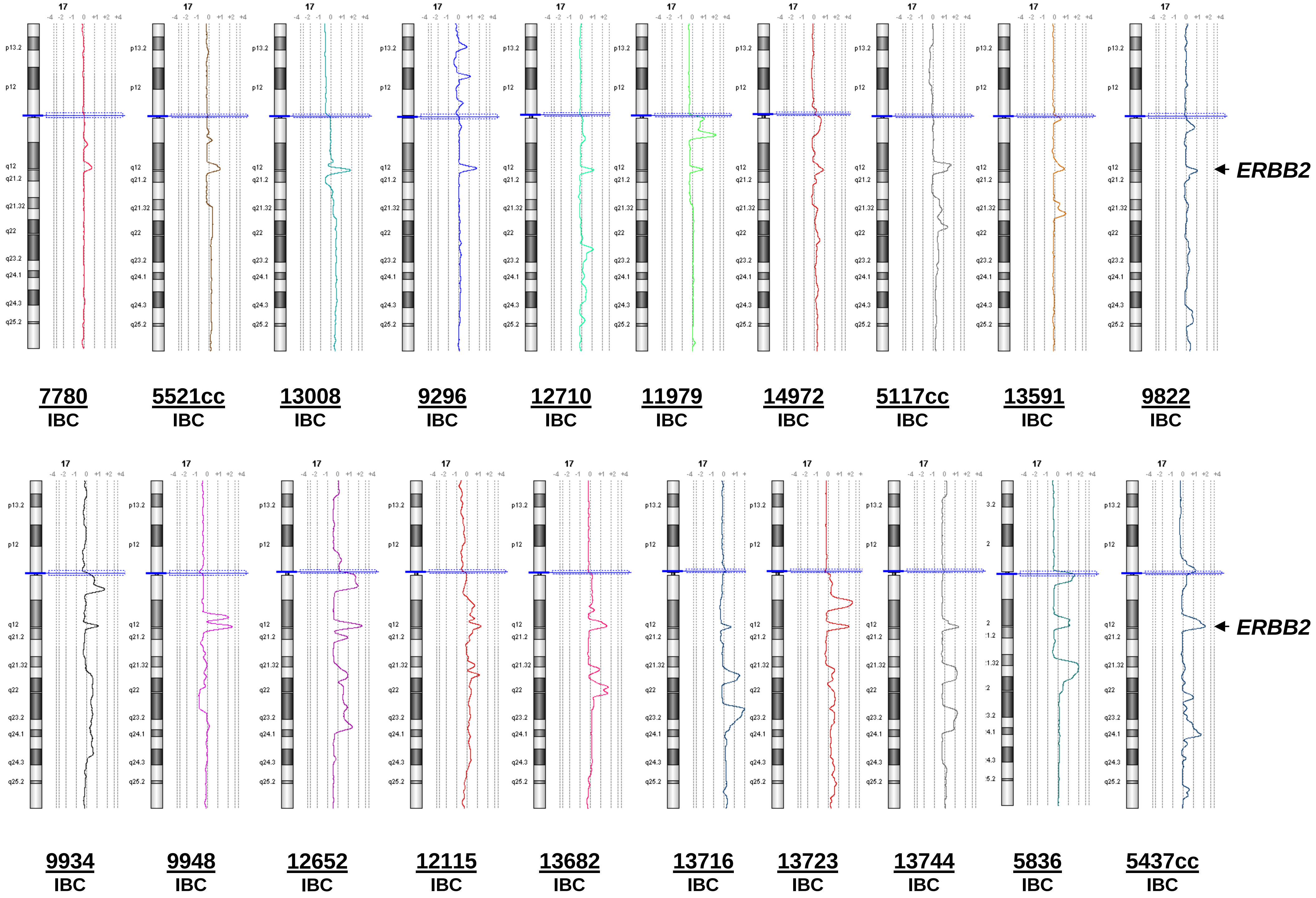
ERBB2
7780
IBC
5521cc
IBC
13008
IBC
9296
IBC
12710
IBC
11979
IBC
14972
IBC
5117cc
IBC
13591
IBC
9822
IBC
ERBB2
9934
IBC
9948
IBC
12652
IBC
12115
IBC
13682
IBC
13716
IBC
13723
IBC
13744
IBC
5836
IBC
5437cc
IBC

Figure S1A

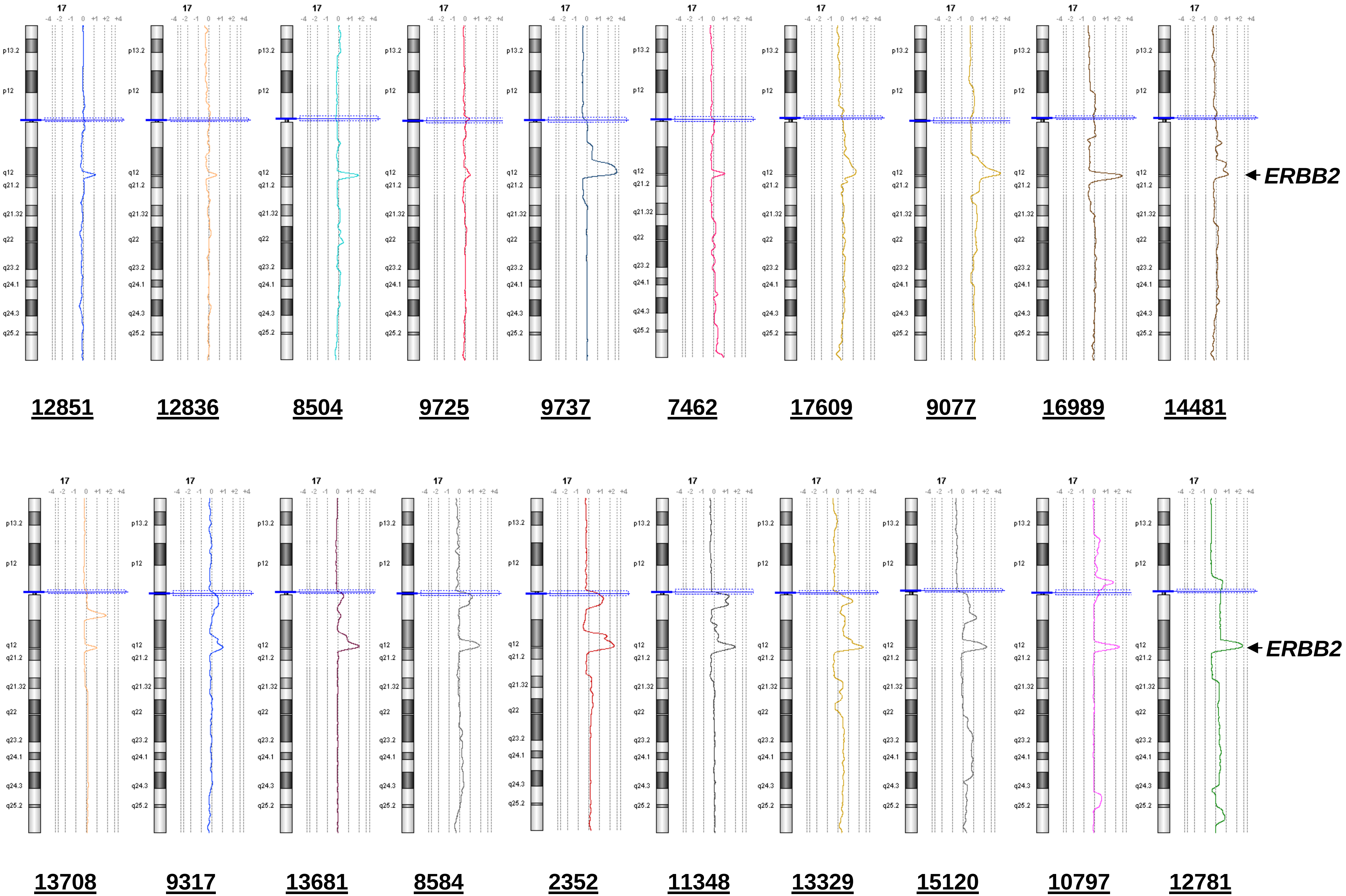
17
-4 -2 -1 0 +1 +2 +4
p13.2
p12
q12
q21.2
q21.32
q22
q23.2
q24.1
q24.3
q25.2
ERBB2
12851
12836
8504
9725
9737
7462
17609
9077
16989
14481
ERBB2
13708
9317
13681
8584
2352
11348
13329
15120
10797
12781

Figure S1B

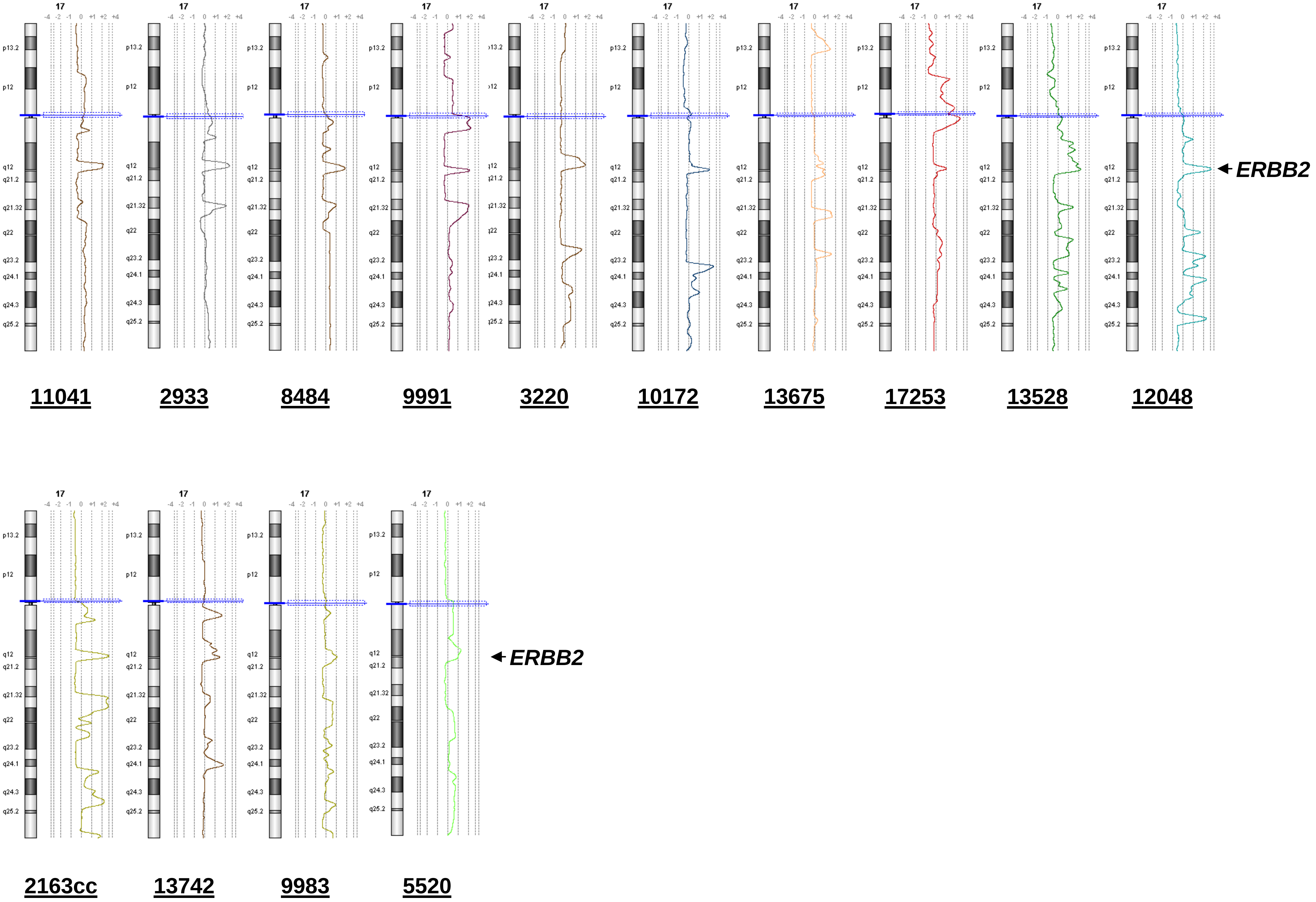

17
-4 -2 -1 0 +1 +2 +4
p13.2
p12
q12
q21.2
q21.32
q22
q23.2
q24.1
q24.3
q25.2
ERBB2
11041
2933
8484
9991
3220
10172
13675
17253
13528
12048
ERBB2
2163cc
13742
9983
5520

Figure S1C

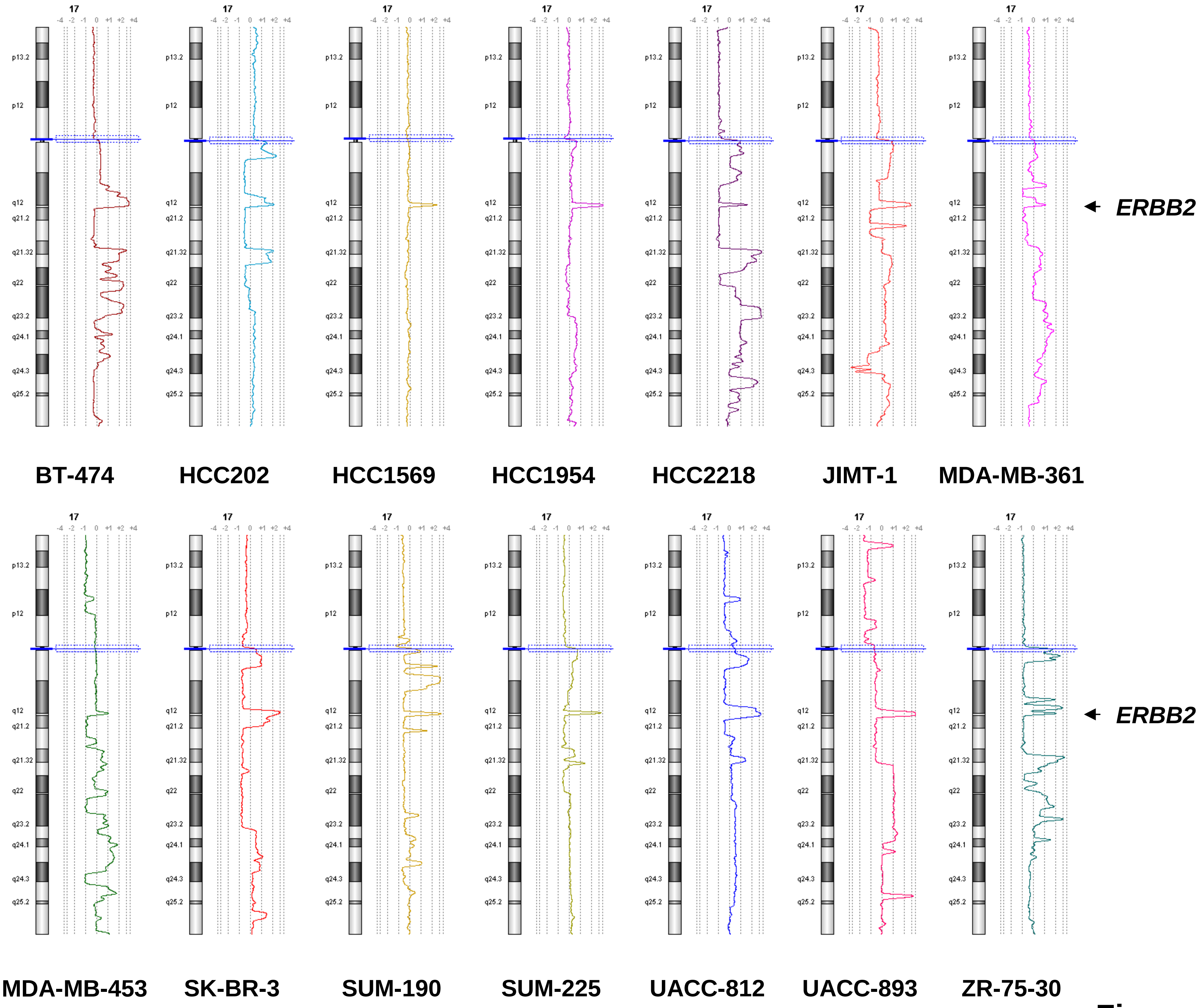
17
p13.2
p12
q12
q21.2
q21.32
q22
q23.2
q24.1
q24.3
q25.2
-4 -2 -1 0 +1 +2 +4
ERBB2
BT-474
HCC202
HCC1569
HCC1954
HCC2218
JIMT-1
MDA-MB-361
ERBB2
MDA-MB-453
SK-BR-3
SUM-190
SUM-225
UACC-812
UACC-893
ZR-75-30

Figure S1D
