# Supplementary figures and images for: Genome profiling of ERBB2-amplified breast cancers
Source: BMC Cancer. 2010 Oct 8;10:539. doi: 10.1186/1471-2407-10-539 (PMC2958950; doi:10.1186/1471-2407-10-539)

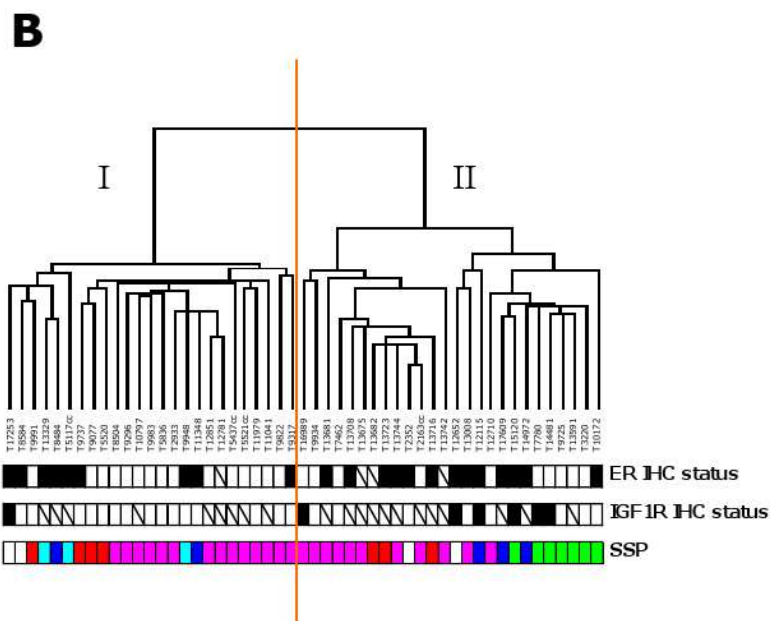

Supplement: Additionnal file 4 — Figure S2: Whole-genome expression profiling of ERBB2-amplified BCs. A - Hierarchical clustering of 51 samples and 13,114 genes/ESTs with significant variation in mRNA expression level across the samples. Each row of the data matrix represents a gene and each column represents a sample. Expression levels are depicted according to the color scale shown at the bottom. Red and green indicate expression levels respectively above and below the median. The magnitude of deviation from the median is represented by the color saturation. The dendrogram of samples (above matrixes) represents overall similarities in gene expression profiles and is zoomed in B. B - Dendrograms of samples. Top, Two large groups of tissue samples (designated I to II) are evidenced by clustering and delimited by the orange solid vertical line (see also Additionnal file 1-Table S7B). Below the dendrogram, Below the dendrogram, from the top to the bottom, name of tumors are given and the two first rows indicate their ER and IGF1R status (black square, ER+ or IGF1R+; white square, ER- or IGF1R-) of patients, while the last row indicates the molecular subtypes. The third row indicates the gene expression molecular subtypes as previously defined [13] using specific colored squares (luminal A: dark blue; luminal B: sky blue; basal: red; ERBB2-like: pink; normal-like: green and not assigned: white). [file 1471-2407-10-539-S4.PDF]

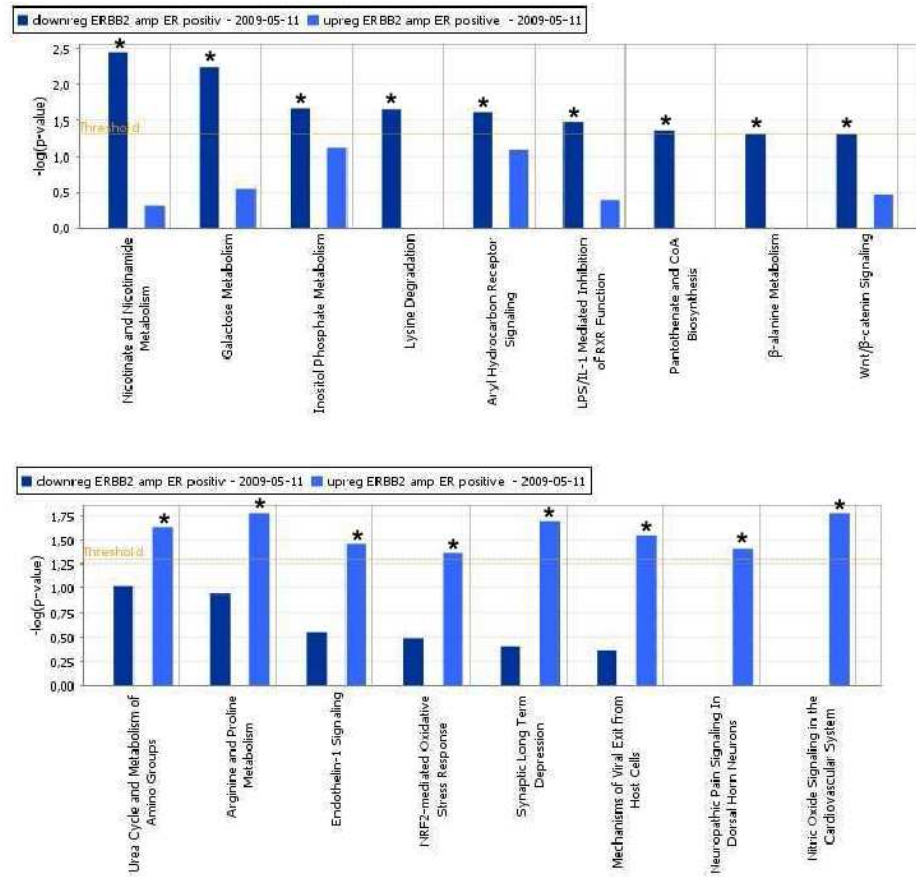

\* Fisher's exact test  $p < 0.05$

Supplement: Additionnal file 5 — Figure S3: Canonical pathways associated with ER+ and ER- ERBB2-amplified BCs. Histograms show canonical pathways associated with either ER+ or ER- ERBB2-amplified BCs. Established by Ingenuity® pathways analysis software, they show relevant proteins (Additionnal file 1-Table S7D) encoded by genes associated with the ER+/ER- ERBB2-amplified BCs molecular signature (Additionnal file 1-Table S7C). Color codes and corresponding legends are indicated in the box located to the left at the top of the figure. [file 1471-2407-10-539-S5.PDF]
